# Polyphenols Extracted from Chinese Hickory (*Carya cathayensis*) Promote Apoptosis and Inhibit Proliferation through the p53-Dependent Intrinsic and HIF-1α-VEGF Pathways in Ovarian Cancer Cells

**DOI:** 10.3390/app10238615

**Published:** 2020-12-01

**Authors:** Zhiping He, Shaozhen Wu, Ju Lin, Ashley Booth, Gary O’Neal Rankin, Ivan Martinez, Yi Charlie Chen

**Affiliations:** 1 The Key Laboratory for Quality Improvement of Agricultural Products of Zhejiang Province, College of Agriculture and Food Science, Zhejiang A & F University, Hangzhou 311300, China;; 2 College of Health, Science, Technology and Mathematics, Alderson Broaddus University, Philippi, WV 26416, USA;; 3 Department of Biomedical Sciences, Joan C. Edwards School of Medicine, Marshall University, Huntington, WV 25755, USA;; 4 Department of Microbiology, Immunology & Cell Biology and WVU Cancer Institute, West Virginia University, Morgantown, WV 26506, USA;

**Keywords:** ovarian cancer, apoptosis, Chinese hickory, p53, polyphenol

## Abstract

Ovarian cancer is the second most common gynecologic cancer with an estimated 13,940 mortalities across the United States in 2020. Natural polyphenols have been shown to double the survival time of some cancer patients due to their anticancer properties. Therefore, the effect of polyphenols extracted from Chinese hickory seed skin *Carya cathayensis* (CHSP) on ovarian cancer was investigated in the present study. Cell viability results showed that CHSP is more effective in inhibiting ovarian cancer cells than normal ovarian cells, with the IC50 value for inhibition of cell proliferation of Ovarian cancer cells (OVCAR-3) being 10.33 ± 0.166 μg/mL for a 24 h treatment. Flow cytometry results showed that the apoptosis rate was significantly increased to 44.21% after 24 h treatment with 20 μg/mL of CHSP. Western blot analysis showed that CHSP induced apoptosis of ovarian cancer cells through a p53-dependent intrinsic pathway. Compared with control values, levels of VEGF excreted by OVCAR-3 cancer cells were reduced to 7.87% with a 40 μg/mL CHSP treatment. Consistent with our previous reports, CHSP inhibits vascular endothelial growth factor (VEGF) secretion by regulating the HIF-1α-VEGF pathway. In addition, we also found that the inhibitory effect of CHSP on ovarian cancer is related to the up-regulation of Phosphatase and tension homolog (PTEN) and down-regulation of nuclear factor kappa-B (NF-kappa B). These findings provide some evidence of the anti-ovarian cancer properties of CHSP and support the polyphenols as potential candidates for ovarian cancer adjuvant therapy.

## 1. Introduction

Ovarian cancer is the second most common gynecologic cancer with an estimated 21,750 new cases and 13,940 mortalities predicted to occur across the United States in 2020 [1]. Globally, there are 239,000 new cases and 152,000 deaths due to ovarian cancer every year [2]. More than two-thirds of all women diagnosed with epithelial ovarian cancer will die from the disease, a fact that has not changed considerably in the last three decades. The lack of effective screening results in 50% to 60% of patients being diagnosed with advanced stage disease [3]. Although treatment for ovarian cancer, including surgery and platinum-based chemotherapy, has improved, the 5- and 10-year survival rates of advanced ovarian cancer stages were only 32% and 15%, respectively [4]. Approximately 80% of ovarian cancer patients initially respond to chemotherapy, but more than 60% of these patients who receive standard treatment will relapse and die due to chemoresistance [5]. Thus, recent research has focused on natural polyphenol compounds, which have been shown to double the survival time of the cancer patients in this century [6].

Nuts are known for their health benefits, including anti-cardiovascular disease and anti-oxidative properties. Recently, a significant inverse association between nut consumption and overall mortality was observed by The National Institutes of Health-American Association of Retired Persons (AARP) Diet and Health Study. This study showed that nut consumption was significantly associated with reduced risk of cancer and cardiovascular, respiratory, infectious, renal, and liver disease mortality [7]. Hardman et al. found that walnut consumption could suppress growth and survival of breast cancers in mice [8]. Recent reports show that phenols have the ability to inhibit tumors [9]. It has been reported that polyphenolic compounds, a large family of natural compounds abundant in nuts, have been associated with a possible reduced risk of colon cancer [10], breast cancer [11] and melanoma [12].

Chinese hickory (*Carya cathayensis*), which has been commercially cultivated for more than 500 years, is a native nut species of Zhejiang Province in China. Our previous research showed that, like other nuts, Chinese hickory nuts are rich in polyphenols (78.28 ± 0.378 mg gallic acid equivalents/g) [13]. However, there is no report on the anti-tumor function of Chinese hickory polyphenols. The flesh of the walnut seed is surrounded by a brown leathery coating, called the skin or the seed coat, which protects the walnut kernel from oxidation and microbial contamination. Component analysis shows that polyphenols are mainly present in the skin of nuts [14,15]. Research on these polyphenols will help the comprehensive utilization of Chinese hickory, but there is little research on the seed skin of Chinese hickory nuts to evaluate anti-tumor activity. The purpose of this study was to investigate the inhibitory effects of Chinese hickory seed skin polyphenols (CHSP) on ovarian cancer cell lines. Furthermore, the inhibitory mechanism of CHSP on ovarian cancer cells was also analyzed by using Western blot and enzyme-linked immunoassay (ELISA).

## 2. Materials and Methods

### 2.1. Cell Culture and Reagents

The human ovarian carcinoma cell lines Ovarian cancer cells (OVCAR-3), A2780/CP70 and human immortalized ovarian surface epithelial cells (IOSE 364), were kind gifts from Dr. Bing-Hua Jiang at Thomas Jefferson University and Dr. Auersperg at the University of British Columbia, respectively. Cells were cultured in RPMI1640 medium (Sigma, St. Louis, MO, USA) incorporating 10% fetal bovine serum (FBS) (Invitrogen, Grand Island, NY, USA). Cells were grown in a humidified incubator containing 5% CO_2_ at 37 °C.

Reagents, Dead Cell Apoptosis Kits with Annexin V AlexaFluor^®^ 488 and propidium iodide (PI), were purchased from Thermo Fisher Scientific (Waltham, MA, USA). Caspase-Glo^®^ 3/7 Assay Systems were purchased from Promega (Madison, WI, USA). Antibodies against Bax and Puma, were purchased from Cell Signaling Technology, Inc. (Danvers, MA, USA). The primary antibodies against p53, HIF-1α, Cleaved PARP, Bad, NF-kappaB, PTEN, and glyceraldehyde-3phosphate dehydrogenase (GAPDH)were purchased from Santa Cruz Biotechnology Inc. (Santa Cruz, CA, USA). Quantikine Human vascular endothelial growth factor (VEGF) Immunoassay kit was purchased from R&D Systems (Minneapolis, MN, USA).

### 2.2. Preparation of CHSP

Chinese hickory (*Carya cathayensis*) seeds were harvested in the mountainous areas of Lin’an, Zhejiang Province, China. Chinese hickory seeds were broken carefully to remove the kernels. All kernels were immersed in a water bath (45 °C) for 30 min. After immersion, the kernels were peeled manually, and the skin was collected in sealed plastic bags at room temperature. Then, the skin was dried in a vacuum oven (DZF-6094A, Shanghai Yiheng Scientific Instrument Ltd. Co., Shanghai, China) at 70 °C for 10 h. Dried skin was ground into powder by a squeezer. Extraction was prepared by macerating 28 g of defatted kernel powder with 280 mL of 80% methanol. The mixture was kept in a rotary shaker overnight and then centrifuged at 3000× *g* for 20 min. The supernatant was carefully separated and evaporated in a rotary evaporator (40 °C) (RE-52, Shanghai Yarong biochemistry Instrument Factory, Shanghai, China). Condensed extracts were lyophilized using a freeze dryer (FD-1A-80, Beijing Boyikang Instrument Experimental Instrument Co., Beijing, China). Finally, 8.25 g of dried methanol extract (powder) of Chinese hickory skin was obtained and stored at 4 °C.

A stock solution of CHSP was prepared in dimethyl sulfoxide (DMSO) at 100 mg/mL and stored at −20 °C. Different concentrations of CHSP were prepared in RPMI-1640 medium for cell treatments, and DMSO was included in the preparations to ensure equal concentrations of DMSO in each treatment.

### 2.3. Determination of Total Phenolic Content and Total Flavonoid Content

Total phenolic content was measured using the Folin-Ciocalteu method with minor modifications. Briefly, 0.01 g CHSP was dissolved in 250 mL methanol. Then, 0.6 mL of the methanol solution of the CHSP was mixed with 3.0 mL of Folin-Ciocalteu reagent (diluted 10-fold) and 2.4 mL of 0.765 mol/L Na_2_CO_3_ kept for 30 min in the dark. Subsequently, absorbance was measured at a wavelength of 765 nm using a spectrophotometer. The total phenolic content (TPC) was determined as micrograms of gallic acid equivalents per gram CHSP. The equation of the calibration curve was *y* = 0.0083*x* + 0.0174, with a correlation coefficient of R^2^ = 0.9977.

Furthermore, 0.01 g CHSP was dissolved in 4 mL methanol. Then, 0.4 mL of the methanol solution of the CHSP was transferred to a 10 mL centrifuge tube and mixed with 0.3 mL of 5% sodium nitrite (*w*/*v*). The mixture was incubated for 6 min. Next, 0.3 mL of 10% aluminum chloride (*w*/*v*) was added and incubated for 6 min. After adding 4 mL of 4% NaOH (*w*/*v*) and incubating in the dark for 15 min, absorbance was measured at 510 nm using ultraviolet (UV)spectrophotometer. The total flavonoid content was determined as micrograms of rutin equivalents per gram CHSP. The equation of the calibration curve was *y* = 0.4872*x* − 0.0038, with a correlation coefficient of R^2^ = 0.9996.

### 2.4. Assessment of Cell Viability

Ovarian cancer cells (OVCAR-3, A2780/CP70) and normal ovarian cells (IOSE 364) were seeded in 96-well plates at a density of 1 × 10^4^/well (medium RPM-1640 + 10% FBS) and incubated at 37 °C for 16 h. Then, the culture medium was removed and cells incubated with different concentrations of CHSP (5–40 μg/mL) or DMSO (as vehicle) for 24 h. After treatment, the cells were washed twice with phosphate-buffered saline (PBS), introduced to 100 μL freshly prepared Aqueous One Solution (MTS tetrazolium compound) (Promega, Madison, WI, USA) in medium, and incubated for 1 h at 37 °C. Cells were then transferred to a microplate reader and the absorption peak was checked at 490 nm. Cell viability was expressed as a percentage of the control.

### 2.5. Apoptosis Analysis

Cells were treated with CHSP (5–20 μg/mL) or DMSO (as vehicle) for 24 h. Then, cells were collected and stained with Annexin V Alexa Fluor^®^ 488 and propidiumiodide (PI) according to the manufacturer’s instructions. Data acquisition and analysis were performed following flow cytometry with accompanying software (FACS Calibur; BD Bioscience, San Jose, CA, USA).

### 2.6. Detection of Caspase-3/7 Enzyme Activities

OVCAR-3 cells were seeded into 96-well plates (1 × 10^4^/well) and incubated overnight at 37 °C. Cells were treated with different concentrations of CHSP (5–20 mg/mL) or DMSO for 4 h. After treatment, the Caspase-Glo 3/7 Assay kit (Promega) was used to detect caspase-3/7 enzymatic activities in OVCAR-3 cells. Enzymatic activities were normalized by total protein levels and were expressed as a percentage of the untreated control.

### 2.7. Western Blot

OVCAR-3 cells (10^6^) were seeded in 60-mm dishes and incubated overnight before treatment of CHSP. The cells were washed once with PBS buffer, lysed in 100 μL mammalian protein extraction reagent including 1 μL Halt Protease, 1 μL phosphatase inhibitor, and 2 μL ethylenediaminetetraacetic acid (EDTA). Cell lysates were separated by 10% Sodium dodecyl sulfate-Polyacrylamide gel electrophoresis (SDS-PAGE), and proteins were transferred to a nitrocellulose membrane using the Mini-Protean 3 System (Bio-Rad Laboratories, Hercules, CA, USA). The membrane was blocked with 5% nonfat milk Tris-buffer containing 0.1% Tween-20 for 1 h (room temperature), and then incubated with the appropriate concentrations of primary and secondary antibodies for the appropriate time. After washing with Tris Buffered saline Tween (TBST) buffer, the Super Signal West Dura Extended Duration Substrate (Pierce) antigen-antibody mixture was added to develop color and luminescence. Protein bands were quantitated with NIH ImageJ software and normalized by GAPDH bands for analysis.

### 2.8. ELISA for VEGF

The effect of CHSP on VEGF protein secretion was analyzed by ELISA with a Quantikine Human VEGF Immunoassay kit (R&D Systems, Minneapolis, MN, USA), targeting VEGF165 in the cell culture supernatant. OVACAR-3 cells (6 × 10^5^) were seeded in 60-mm cell culture dishes and grown for 16 h at 37 °C before treatment with various concentrations of CHSP (5–40 μg/mL) or DMSO (as vehicle) for 24 h. Culture supernatants were collected for the VEGF assay. The inhibition of VEGF protein secretion was expressed as a percentage of the control. Cell lysates were also assayed for total protein levels using a BCA protein assay kit (Pierce, Rockford, IL, USA) to adjust VEGF levels.

### 2.9. Transient Transfection and Luciferase Assay

OVCAR-3 cells (1 × 10^4^ cells/well) were seeded onto 96-well plates and incubated overnight before transfection with HIF-1α plasmids (Addgene, Cambridge, MA, USA). Cells were transfected with VEGF luciferase reporter (0.05 μg) and HIF-1α plasmids (0, 0.0625, 0.125, and 0.25 μg) or SR-a (as vehicle) plasmids using 0.6 μL of jetPRIME reagent (VWR, West Chester, PA, USA) for 4 h. After transfection, all of the cells were treated with CHSP (10 μg/mL) or vehicle for 16 h. The cells were harvested and analyzed for luciferase activity and total protein levels using a BCA Protein Assay Kit (Pierce), and the activities of the VEGF reporter were normalized to corresponding total protein levels for statistical analysis.

### 2.10. Statistical Analysis

Each experiment was conducted at least three times. The experimental data were expressed as means ± standard deviation (SD). The experimental results were analyzed by a post hoc test (Duncan test) and one-way analysis of variance (ANOVA) to test differences between each treatment and control. A *p*-value of <0.05 was considered statistically significant.

## 3. Results

### 3.1. Determination of the Total Phenolic Content and Total Flavonoid Content of CHSP

Phenolic compounds of nuts are generally concentrated in the brown skin as soluble free and bound forms [16]. In this research, the total phenolic content and total flavonoid content of CHSP were determined. The results showed that the total phenolic content and total flavonoid content of CHSP were 767.9 ± 11.2 micrograms of gallic acid equivalents per gram CHSP and 511.6 ± 9.6 micrograms of rutin equivalents per gram CHSP.

### 3.2. Inhibitory Effect of CHSP on Proliferation of Ovarian Cancer Cells

**Figure 1.**
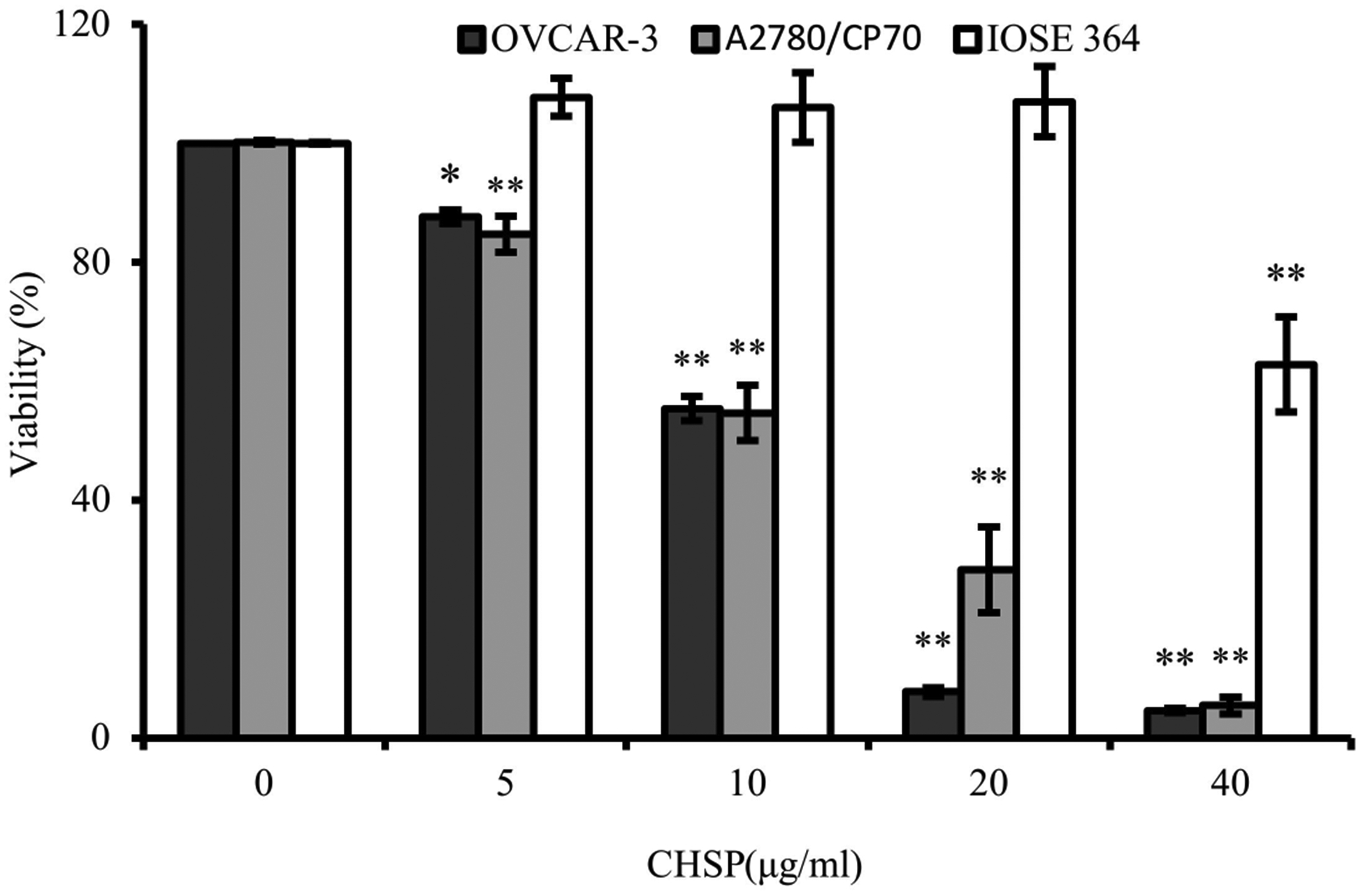
Effect of Chinese hickory seed skin Carya cathayensis (CHSP) on cell viability of ovarian cancer cells (OVCAR-3, A2780/CP70) and normal ovarian cells (IOSE 364). * *p* < 0.05 as compared to control. ** *p* < 0.01 as compared to control.

To assess cell viability, the CellTiter 96^®^ Aqueous One Solution Cell Proliferation Assay was performed. As shown in Figure 1, CHSP showed significant inhibitory effect on two ovarian cancer cell lines OVCAR-3 and A2780/CP70 in a concentration-dependent manner. When the CHSP concentration increased from 5 to 40 μg/mL, the cell viability of OVCAR-3 was decreased from 87.6% to 4.7% and A2780/CP70 from 84.7% to 5.5%. The IC50 value OVCAR-3 and A2780/CP70 were found to be 10.33 ± 0.17 and 11.43 ± 0.76 μg/mL. At 40 μg/mL, the cell viability of CHSP on IOSE364 was 62.4%, which is much larger than the value of OVCAR-3 and A2780/CP70 cells.

### 3.3. CHSP Induces Apoptosis in Ovarian Cancer Cells through the p53-Dependent Intrinsic Pathway

**Figure 2.**
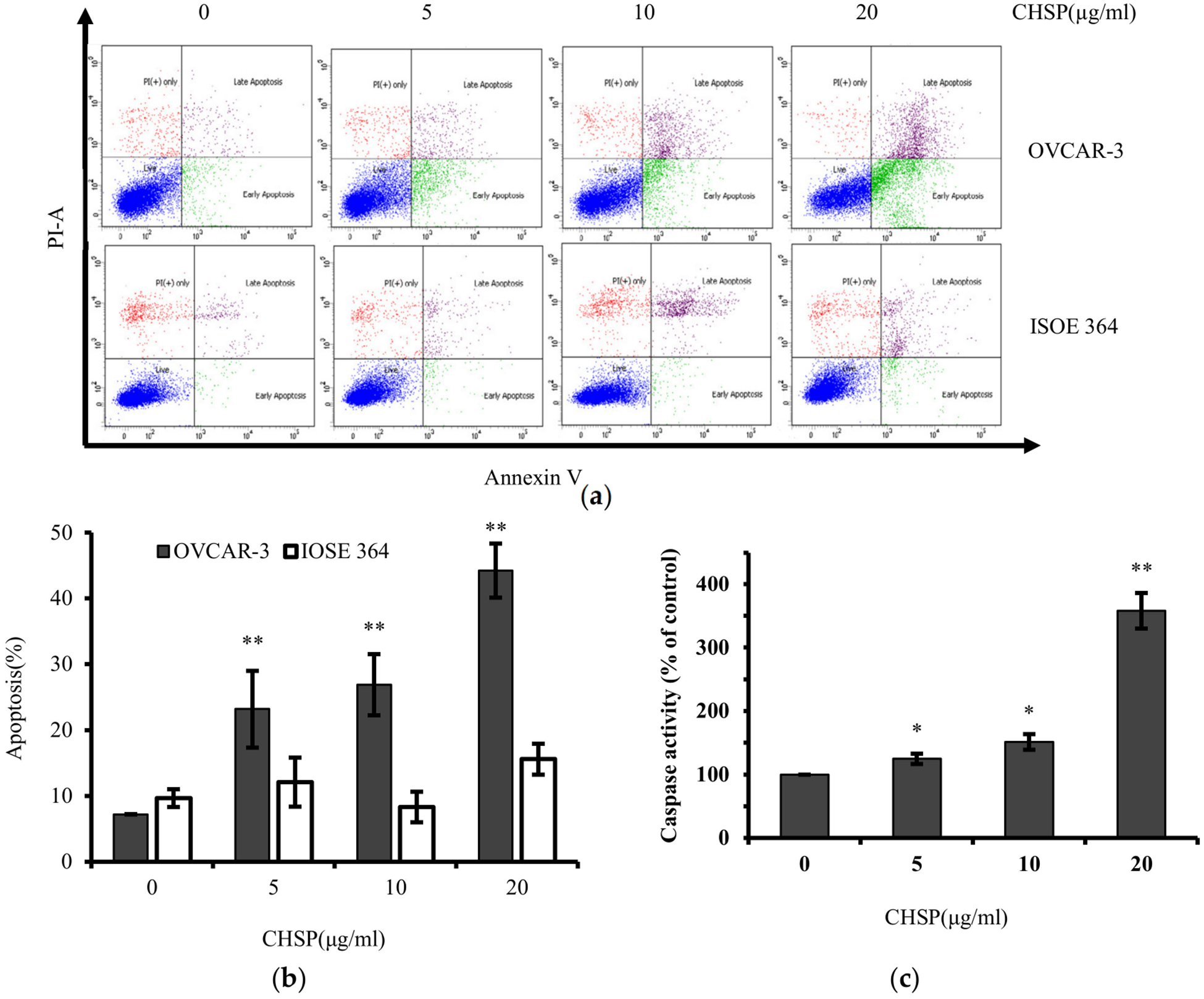
CHSP induced apoptosis in ovarian cancer cells (OVCAR-3) and normal ovarian cells (IOSE 364). (**a**) CHSP induced apoptosis was analyzed by flow cytometry. Cells treated with CHSP for 24 h were collected, stained with PI and Annexin V, and analyzed by flow cytometry. (**b**) Calculation of the apoptotic rate of the data in A. (**c**) Induction of caspase by CHSP. OVCAR-3 cells were treated with CHSP for 4 h, and caspase-3/7 enzymatic activity was determined using a Caspase-Glo 3/7 assay kit. * *p* < 0.05 as compared to control. ** *p* < 0.01 as compared to control.

**Figure 3.**
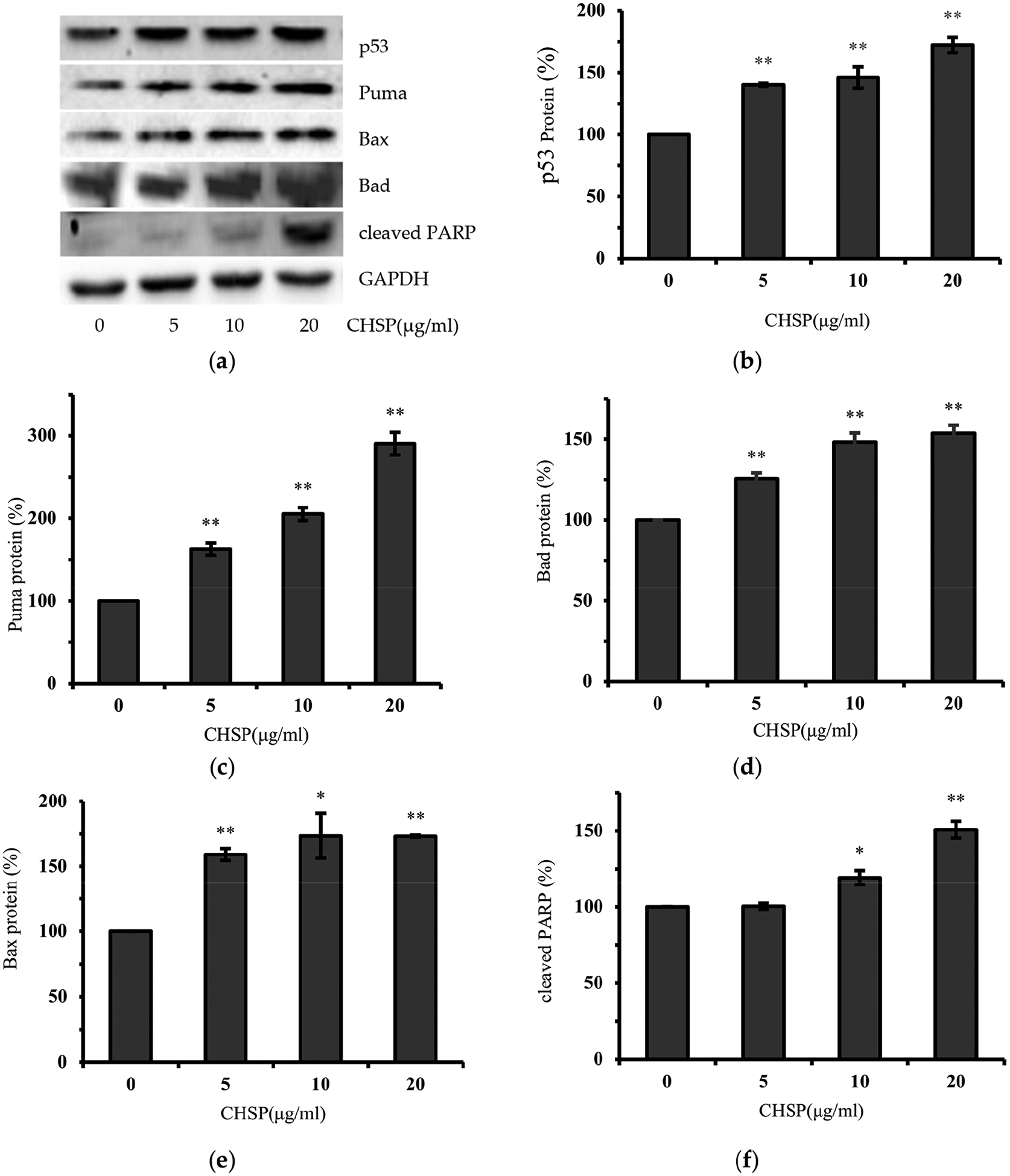
CHSP activated the p53-dependent intrinsic pathway. (**a**) CHSP increased the levels of Puma, Bax, Bad, cleaved PARP, and p53 in OVCAR-3 cells. (**b**) p53. (**c**) Puma. (**d**) Bax. (**e**) Bad. (**f**) cleaved PARP. * *p* < 0.05 as compared to control. ** *p* < 0.01 as compared to control.

Annexin V and propidium iodide (PI) staining was performed for flow cytometry analysis. As shown in Figure 2, an increase in cell apoptosis in OVCAR-3 cells was induced in a concentration-dependent manner after CHSP treatment. At 20 μg/mL of CHSP, the apoptosis rate was 44.21%. Similar to the results of cell viability above, there was also no significant apoptosis rate of normal cells (IOSE364). In this study, caspase-3/7 enzymatic activities were detected by using a Caspase-Glo 3/7 Assay kit. As shown in Figure 2c, compared to controls, the caspase-3/7 enzymatic activities were maximally increased to 3.57-fold in OVCAR-3 cells when treated with 20 μg/mL CHSP for 24 h. Moreover, it was identified that there is an up-regulation in pro-apoptotic proteins including Bax (1.73-fold), BAD (1.53-fold), cleaved PARP (1.5-fold), and Puma (2.9-fold), shown in Figure 3.

### 3.4. CHSP Inhibits Ovarian Cancer Cell Proliferation via the HIF-1α/VEGF Pathway

**Figure 4.**
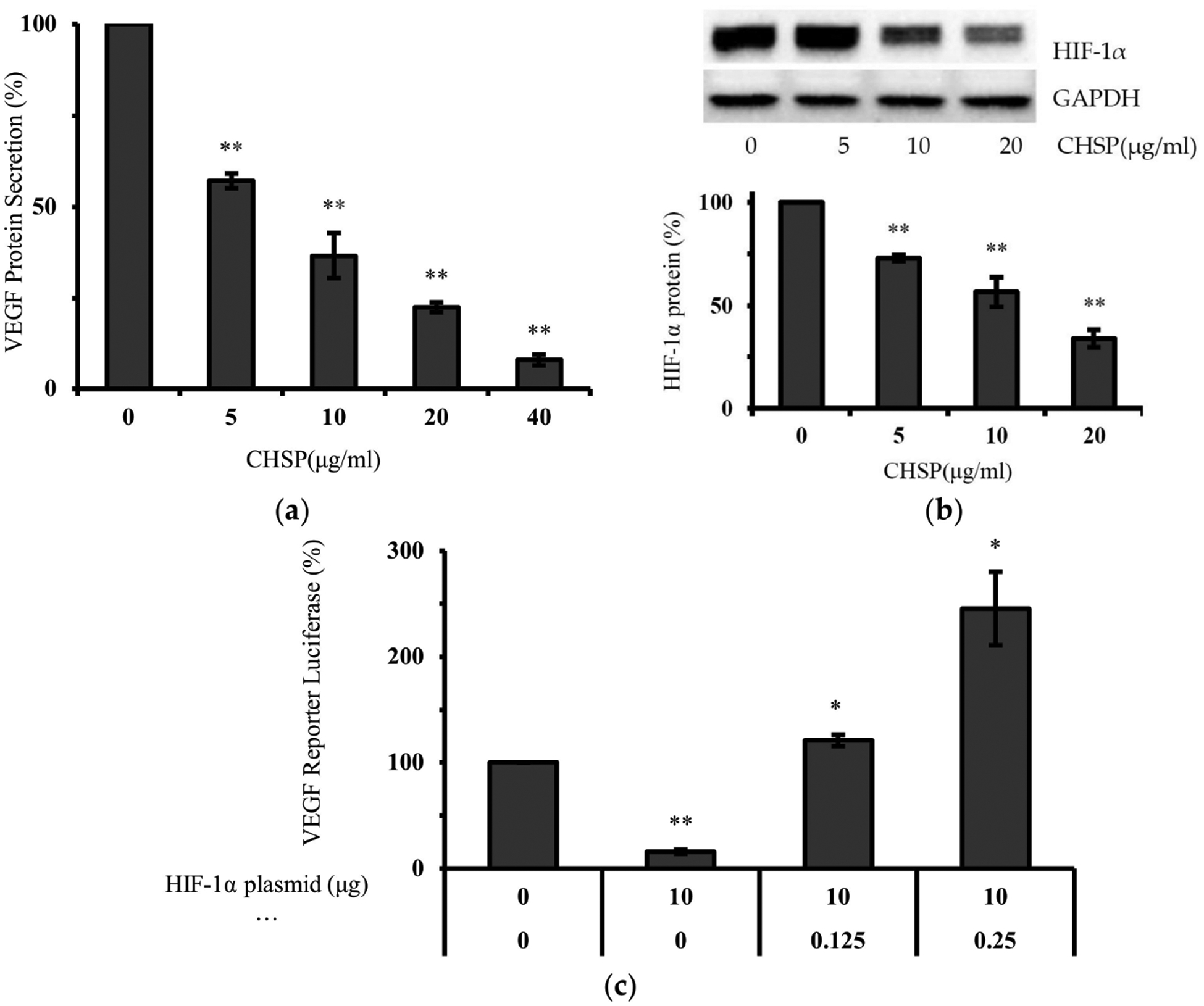
CHSP inhibited cell proliferation via the HIF-1α/VEGF pathway in OVACAR-3 cells. (**a**) Cells were treated with CHSP for 24 h. Culture supernatants were collected for the VEGF assay by ELISA with a Quantikine Human VEGF Immunoassay kit. (**b**) CHSP decreased the levels of HIF-1α and HIF-1α bands were quantitated with NIH Image J software for analysis. (**c**) Cells were transfected with VEGF luciferase reporter and HIF-1α plasmids. After transfection, all the cells were treated with or without CHSP (10 μg/mL) for 16 h and the luciferase activity was determined. * *p* < 0.05 as compared to control. ** *p* < 0.01 as compared to control.

As shown in Figure 4a, levels of VEGF excreted by OVCAR-3 cells were inhibited to 57.12, 36.66, 22.55 and 7.87% by 5, 10, 20 and 40 μg/mL CHSP treatments, respectively. Also shown in Figure 4, HIF-1α expression was significantly inhibited by CHSP treatment. Compared to the control level, HIF-1α expression at 20 μg/mL of CHSP decreased by 65.8%. Shown in Figure 4b, this inhibition of VEGF was significantly reversed by forced expression of HIF-1α protein.

### 3.5. CHSP Up-Regulates the Expression of PTEN and NF-Kappa B Protein in Ovarian Cancer Cells

**Figure 5.**
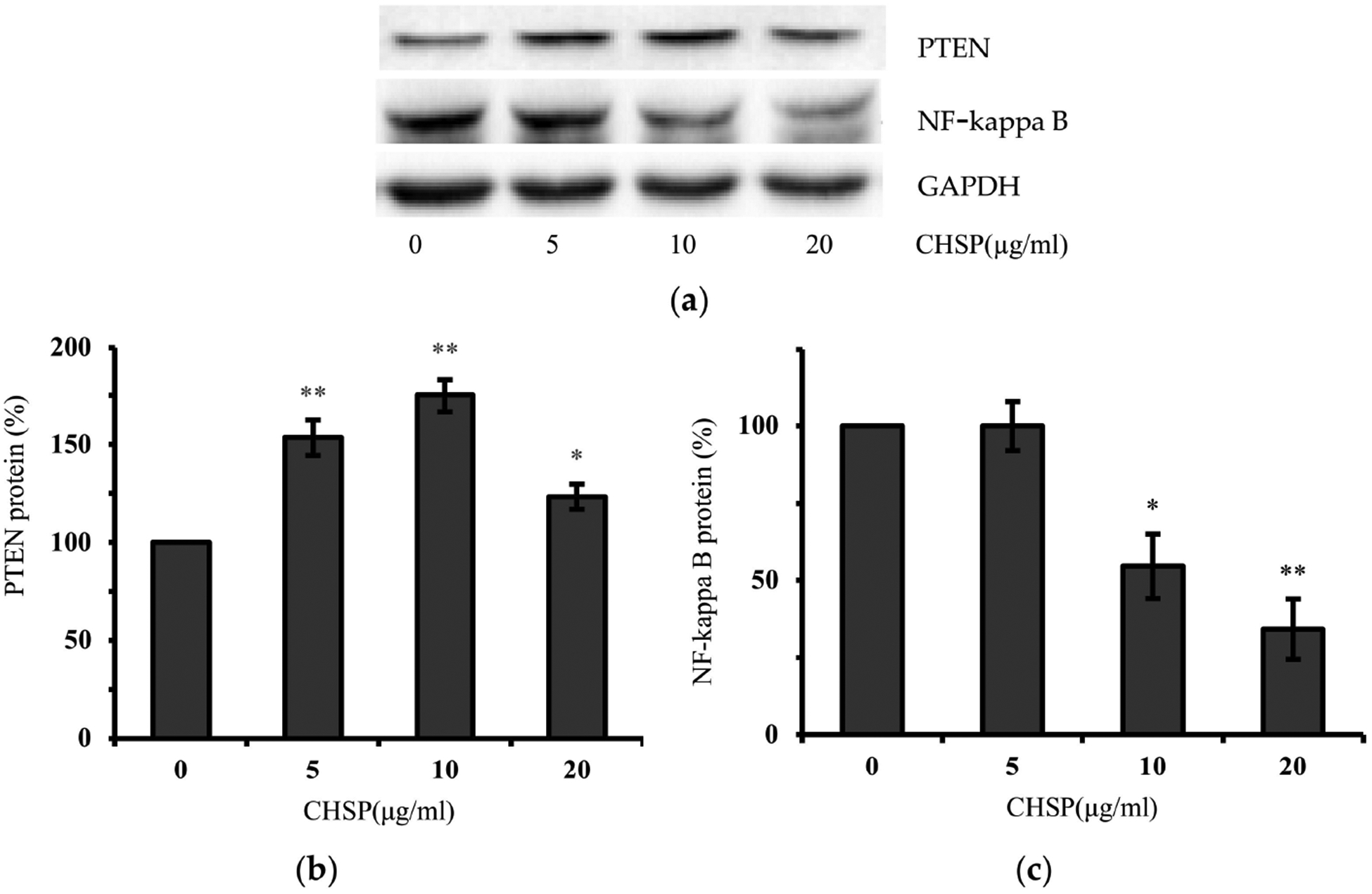
CHSP up-regulated the expression of PTEN and down-regulated NF-kappa B protein in OVACAR-3 cells. (**a**) CHSP increased the levels of PTEN and decreased the levels of NF-kappa B in OVCAR-3 cells. (**b**) PTEN. (**c**) NF-kappa B. * *p* < 0.05 as compared to control. ** *p* < 0.01 as compared to control.

As shown in Figure 5, the expression of PTEN was increased by treatment of OVACAR-3 cells with CHSP. Compared to control values, PTEN expression at 10 μg/mL of CHSP increased by 75.1%. Represented in Figure 5, the expression of NF-kappa B was down-regulated after CHSP treatment. In addition, NF-kappa B expression at 20 μg/mL of CHSP decreased by 65.8% as compared to the control level.

## 4. Discussion

Polyphenols, found in nuts, fruits, vegetables, grains, spices, tea, coffee, and wine, are known for their powerful antioxidant properties [17]. Epidemiological studies on the anticancer effects of natural polyphenols have yielded mixed results due to the difficulty of measuring dietary intake of these compounds [18]. This observation suggests that even more research, such as the current study, is needed to evaluate the therapeutic utility and safety of polyphenolic compounds. CHSP is more effective in inhibiting ovarian cancer cell proliferation than paeonol [19] or extracts of strawberry, Korean raspberry, and mulberry [20]. The lower cell viabilities demonstrate that CHSP has high potential for the prevention and therapy of ovarian cancer. Cell viability with 40 μg/mL of CHSP on IOSE364 was much larger than that of OVCAR-3 and A2780/CP70 cells, indicating that the inhibitory effects of CHSP on normal ovarian cells were less than that on the ovarian cancer cells. Therefore, CHSP selectively inhibits cancer cells while having significantly weaker inhibitory effects on normal ovarian cells.

Next, we assessed whether CHSP treatment in ovarian cancer cells resulted in cellular apoptosis. CHSP was shown to promote apoptosis in OVCAR-3 cells. There was also no significant apoptosis induction in IOSE364 by CHSP, further confirming the selective inhibitory effects of CHSP on ovarian cancer cells compared with normal ovarian cells. Apoptosis is a form of programmed cell death [21]. The intrinsic (mitochondria-mediated) and extrinsic (receptor-mediated) pathways are two major apoptotic pathways. The up-regulation in pro-apoptotic proteins by CHSP are related to the mitochondrial apoptosis pathway [22,23]. Therefore, mitochondrial apoptosis pathway is likely to be the main reason for CHSP-induced apoptosis. However, some studies in breast cancer patients found that daily intake of natural polyphenols may stimulate cancer growth due to the interaction between the polyphenolic compound and endogenous steroid hormones [24].

Our previous work reported that polyphenols, prodelphinidins [25], theaflavin-3′-gallate [26] and galangin [27], induced apoptosis in ovarian cancer cells through the activation of p53 protein. To determine whether p53 is involved in CHSP-induced apoptosis of ovarian cancer cells, the expression of p53 protein was detected by Western blot. Consistent with previous results from other purified polyphenols, after CHSP treatment, p53 protein in OVACAR-3 cells was also up-regulated. This result indicates that p53 plays an important role in the apoptosis of ovarian cancer cells induced by polyphenols and CHSP can induce apoptosis through the p53-dependent pathway.

VEGF plays an important role in ovarian cell proliferation and angiogenesis [28,29]. To assess the effect of CHSP on proteins affecting cell proliferation, levels of VEGF were examined. Numerous studies have shown that HIF-1α is a primary regulator of VEGF production [30–32]. Thus, we first examined the influence of CHSP treatment on HIF-1α expression. To confirm that HIF-1α expression is not only regulated by CHSP treatment but also plays a role in the inhibition of VEGF secretion by CHSP, OVCAR-3 cells were transfected with the VEGF-promoter reporter, together with HIF-1α plasmids. The higher the expression of HIF-1α, the higher the increase in the expression of the VEGF reporter. These results demonstrated that CHSP inhibits VEGF production through a HIF-1α-dependent pathway. This finding is consistent with our previous report that gallic acid, an important polyphenol found in Chinese hickory, inhibits the secretion of VEGF from ovarian cancer cells through the HIF-1α pathway [33].

Several phytochemicals, which contain polyphenols, have shown promising results for cancer therapeutics due to their inhibition of VEGF production in ovarian cancer cells [34]. However, all polyphenols have varying effects on cancer cell growth [35]. Our previous research showed that gallic acid, a polyphenolic compound, inhibits the secretion of VEGF by up-regulating the expression of PTEN protein. Out of eight tested phenolic compounds, gallic acid exhibited the most significant inhibitory effect on OVCAR-3 cells [36]. Varela-Rodriquez et al. reported that gallic acid reduced cell viability by 50% in OVCAR-3 cells at 43 μg/mL [37]. Liao et al. also reported anticancer effects of gallic acid, showing that gallic acid regulated cell proliferation of bladder cancer cells via the PI3K/AKT and MAPK/ERK pathway, as well as inhibited bladder cancer cell growth, invasion, and migration through fatty acid synthase inhibition [38]. Not only is gallic acid an effective anticancer agent individually, it was also found to enhance the effects of cisplatin in the inhibition of cancer cell proliferation and induction of apoptosis in non-small lung cancer [39].

Several studies reported that PTEN and NF-kappa B are associated with apoptosis and anti-proliferation of cancer cells. Over-expression of PTEN can effectively promote apoptosis of liver cancer cells [40], while the loss of PTEN can inhibit apoptosis in ovarian cancer cells [41]. Xue et al. reported that PTEN inhibition reduced cell apoptosis and enhances proliferation in human umbilical vein endothelial cells [42]. PTEN expression was up-regulated with CHSP treatment which shows a possible correlation with the increase of apoptosis and anti-proliferation.

The NF-kappa B family of eukaryotic nuclear transcription factors exists widely in cells from insects to humans and is involved in cell differentiation, cell proliferation, apoptosis, adhesion, and inflammatory responses. When various external signals act on cells, activated NF-kappa B enters the nucleus and performs its functions. Some reports showed that inhibition of NF-kappa B signal pathways can promote apoptosis [43]. Previously, it was shown showed that plumbagin inhibits tumor cell proliferation of gastric cancer through the NF-kappa B pathway [44]. NF-kappa B was down-regulated with CHSP treatment, showing that similar to PTEN, promotion of apoptosis and anti-proliferation in ovarian cancer cells may also be attributed to the down-regulation of NF-kappa B protein.

## 5. Conclusions

Cell proliferation inhibition and apoptosis induction by CHSP in ovarian cancer cells were investigated in this study. The IC50 of inhibition on cell proliferation of OVCAR-3 cells was found to be 10.33 ± 0.166 μg/mL. Flow cytometry results showed that the apoptosis rate was significantly increased after CHSP treatment. A 20 μg/mL treatment of CHSP induced ovarian cancer cell apoptosis of 44.21%. Western blot analysis showed that CHSP induced apoptosis of ovarian cancer cells through a p53-dependent intrinsic pathway. In this research, the anti-proliferation of ovarian cancer cells by CHSP was also detected. Compared with control values, levels of VEGF excreted by OVCAR-3 cells were inhibited to 7.87% at 40 μg/mL of CHSP. Consistent with our previous reports, CHSP inhibited VEGF secretion by regulating the HIF-1α-VEGF pathway. In addition, we also found that the inhibitory effect of CHSP on ovarian cancer was related to the up-regulation of PTEN and down-regulation of NF-kappa B.
